# General practitioners’ perceptions of distributed leadership in providing integrated care for elderly chronic multi-morbid patients: a qualitative study

**DOI:** 10.1186/s12913-022-08460-x

**Published:** 2022-08-25

**Authors:** Harald Braut, Olaug Øygarden, Marianne Storm, Aslaug Mikkelsen

**Affiliations:** 1 University of Stavanger Business School, Stavanger, Norway; 2 NORCE Norwegian Research Centre AS, Stavanger, Norway; 3 Department of Public Health, Faculty of Health Sciences, University of Stavanger, Stavanger, Norway; 4 Faculty of Health Sciences and Social Care, Molde University College, Molde, Norway

**Keywords:** Distributed leadership, Shared leadership, Integrated care, Multimorbidity, Home care

## Abstract

**Background:**

Distributed Leadership (DL) has been suggested as being helpful when different health care professionals and patients need to work together across professional and organizational boundaries to provide integrated care (IC). This study explores whether General Practitioners (GPs) adopt leadership actions that transcend organizational boundaries to provide IC for patients and discusses whether the GPs’ leadership actions in collaboration with patients and health care professionals contribute to DL.

**Methods:**

We interviewed GPs (*n* = 20) of elderly multimorbid patients in a municipality in Norway. A qualitative interpretive case design and Gioia methodology was applied to the collection and analysis of data from semi-structured interviews.

**Results:**

GPs are involved in three processes when contributing to IC for elderly multimorbidity patients; the process of creating an integrated patient experience, the workflow process and the process of maneuvering organizational structures and medical culture. GPs take part in processes comparable to configurations of DL described in the literature. Patient micro-context and health care macro-context are related to observed configurations of DL.

**Conclusion:**

Initiating or moving between different configurations of DL in IC requires awareness of patient context and the health care macro-context, of ways of working, capacity of digital tools and use of health care personnel.

**Supplementary Information:**

The online version contains supplementary material available at 10.1186/s12913-022-08460-x.

## Background

The aging population and the growing numbers of frail elders living at home has made the provision of integrated health care services a challenge in high-income countries [1, 2]. Research has shown that collective forms of leadership that extend across people and organizations can help align and coordinate health and care service networks with the needs of complex patients [3, 4]. The aim of this study is therefore to investigate the leadership actions General Practitioners (GPs) adopt to collaborate with patients and other health care professionals to provide integrated care (IC) for complex patients, and whether this form of leadership can be understood to be “leadership across the system”.

IC can be defined in different ways [5, 6]. We will, however and for the purpose of this study, define IC as “initiatives that seek to improve outcomes for those with (complex) chronic health problems and needs by overcoming issues of fragmentation through linkage or coordination of services of different providers along the continuum of care” [7].

IC requires leadership across sectors and institutions, all with different funding streams and information and communication systems, which can create barriers [3, 8]. A review of IC frameworks found that concepts of leadership and governance are addressed by the majority of frameworks [9]. Research into whether and how such leadership plays out in everyday practice is, however, limited [4]. What are the underlying complexities of effective implementation and what are the causes of the outcomes observed, beyond the statement that “leadership matters” [10]?

Leadership is commonly defined as “a process whereby an individual influences a group of individuals to achieve a common goal [11]”. The focus in Distributed Leadership (DL) is, however, on processes in which two or more people (not necessarily all members of an organization) display leadership [12, 13]. DL therefore describes the capacity of an organization and individuals to share responsibility and competence in a given situation and within the environment in which they operate. DL is based on the view that different types of expertise are an advantage in the management of complex tasks, which cannot all be dealt with by one health care professional alone.

In this study DL is understood as a holistic, social process and group attribute [12]. Leadership is applied where the required expertise and motivation is located, this form of leadership being less affected by organizational roles and structures. The health care sector has been described as a “special arena” for DL [14], in which it is suggested professional and institutional interests play a more significant role [15].

Pure DL is characterized by the presence of both concertive action and conjoint agency [15–17]. Concertive action is found in situations where there is (1) spontaneous collaboration between stakeholders who each contribute their expertise to the solving of a problem, (2) a “shared role space” that emerges, in which two or more people share a mutual understanding, a trust and a dependency on each other, and (3) an institutionalization of the leadership practices that result from the learning acquired from (1) and (2) [18]. Conjoint agency means that a “shared mind” has been developed, and that leadership practice directions align.

This study contributes to the literature on IC by investigating whether and how GPs adopt leadership actions that transcend organizational boundaries when providing IC to elderly patients with multimorbidity. We examine the structures and the tools used in interactions between GPs and other health care professionals, between for example hospital specialists, physiotherapists, home care nurses and municipality emergency room staff. We also examine whether the GP’s way of working with health care professionals and the GP’s actions contribute to DL in the treatment and care process. The research questions of this paper are, based on this, therefore; What type of leadership actions do GPs adopt in the collaboration with other health care professionals and the patient in order to provide IC? Do these leadership actions contribute to DL? and Can the collaboration between GPs, patients and other professionals be characterized as DL?

## Methods

### Setting

This study is part of the research project Leadership and Technology for Integrated Health Care Services conducted in a Norwegian municipality of approximately 80,000 residents.

In the municipality, patients receive primary health care from a variety of GPs during office-hours (Monday to Friday) and acute and essential treatment from the local emergency room open 24 hours a day. Patients are diagnosed and managed in GP practices or the local emergency room and referred to the local inter-municipal acute ward or the nearby regional university hospital when required. Home care services are organized into district units staffed by nurses and aides, who provide personal care, nursing, medical services, and terminal care.

Home care nurses and GPs communicate via digitally provided text correspondence, telephone, or meetings in office hours, and nurses and GPs receive copies of electronic health care records from the emergency room and of discharge notes after hospitalization. GPs can communicate digitally and via phone with specialist doctors at the hospital.

The Norwegian Coordination Reform of 2012 [19, 20] and the National Health and Hospital Plan 2020–2023 [21, 22] reflects challenges that are common to health care systems in Western countries with aging populations [3, 23]. The reforms recognizes that the number of elderly people is increasing, and aims to create a more cohesive and coordinated health care service [19, 20], adapted to patients level of health literacy, and with patients as active participants in their own health and treatment [21, 22]. The Norwegian Coordination Reform primarily introduced economic incentives and legal measures to transfer tasks from specialist health care to the municipalities, to strengthen preventive care in the municipalities, and to streamline specialist health care services to secure the best possible use of health care resources [19, 20].

Concerning the potential role of DL in IC in the Norwegian setting, analysis of Norwegian reform initiatives have emphasized that a “mediating structure” is lacking in the Norwegian health care system where primary and secondary health care services are physically separated, have different professional cultures and belong to different administrative levels [24]. Recently, health care communities have been introduced to ensure GP and user representation at all organizational levels, and to support overarching goals of creating the patients’ health care service within a sustainable health care system [25]. Patients should, in this system, be listened to, should be enabled to take active part in health and treatment, and resources should be equally distributed between patients based on the common values of fairness, equality and human dignity [22].

### Design, recruitment, participants and ethics

We apply the Gioia methodology, a systematic approach that allows researchers to study dynamic phenomena and processes with “qualitative rigor”, to this interpretive case study [26]. As the Gioia methodology follows an interpretive logic where social reality is viewed as socially constructed and made meaningful by our understanding of events, the research group considered the methodology to be well-suited to the study of DL as a group-level social process.

The Gioia methodology is inductive, and primarily involves reporting the voices of knowledgeable informants (data) in tandem with the voice of researchers (theory) [26]. This can generate data-to-theory connections, and improved understanding of the processes under study in ways that “give meaning to both people living that experience and social scientific theorizing” [27].

In practice, the methodology is a three-step analytical procedure where the first step of coding is informant-centric and consists of revealing first orders codes, which are derived from the words, phrases and lived experience of individual participants [28, 29] and grouped together into first order concepts. The second stage, which is researcher-centric, consist of combining the identified first order concepts into second-order themes that relate to existing theory and research [26]. The third and last stage of the analytical procedure is to refine the second-order themes and identify the overarching aggregate dimensions emerging from the second order themes [26].

Being informant-centric the Gioia-methodology is well suited to assist researchers in grounded theory development. However, as commonly observed in research literature, our professional backgrounds and familiarity with previous research on DL and IC disposes us to apply the Gioia methodology in more “abductive” than “inductive” ways [30]. The background of the project group members are complementary and multidisciplinary, members possessing work and academic experience from human resources, leadership, medicine, and nursing.

The administrative leader of the Division of health and social care services in the municipality was contacted to gain permission to conduct the study in the municipality. Formal contracts of cooperation were entered into with the municipality for the duration of the research project period (2019–2020). The chief medical officer in the municipality was contacted to gain access to the GPs in the municipality.

A total of twenty GPs were recruited. A sample size of 15–30 participants is judged sufficient in qualitative research and data saturation was reached after about 15 interviews [31, 32]. GPs were approached directly (*n* = 24) by phone, at their practice or introduced to the research project via a professional meeting were the majority of the 70 GPs in the municipality attended. Twelve of these GPs identified patients from their practices who were recruited in tandem with the GP. As it was acknowledged that patients were frequently referred to the inter-municipal acute ward from the local emergency room, we approached the leader of the inter-municipal acute ward who established contact with two nurses who recruited 8 patients from the acute ward. We approached the GPs of these patients after the patient had consented to participate in the study. We approached 24 patients, 20 ultimately participating in the project. When both the patient and his or her GP consented to participate in the study, administrative personnel from the municipality health care services helped identify and recruit the home care nurse most familiar with the patient.

The patients, their GPs and home care nurses were connected to each other as a result of working in groups of three in the municipality. This means that this represents a purposive sampling of participants. We recruited patients who were at least 65 years of age, had been diagnosed with two or more medical conditions [33], received treatment with at least four medications, were enrolled with home care services and had been hospitalized within the last 12 months. Patients suffering from severe dementia or other medical conditions that made recruitment or participation difficult were excluded.

The research project was exempt from formal review by the Regional Committee for Medical and Health Research Ethics in Norway (ref. no. 2019/1138) as the research project was considered health service research that did not intend to generate new knowledge about health and disease. The research project was registered and conducted in accordance with the protocol of the Norwegian Centre for Research Data (ref. no. 228630). Written leaflets and oral information were provided to the municipality acute ward, GPs and nurses, to ensure that all recruited patients understood the research related information. All participants were informed that they could access the data collected and that they could withdraw from the study. Informed participation consents were obtained from GPs, and informed consents and disclosures of confidentiality were obtained from their patients, all being obtained prior to the GP interviews. All informants were assigned a study number to secure confidentiality. One of the 20 patients who approved disclosure of confidentiality before the GP and home care nurse interviews, later withdrew their consent of disclosure, but did not withdraw their participation. The data was therefore adjusted accordingly.

### Data collection

Semi-structured interviews with GPs were conducted by two PhD students (HMH, HB). The two are cumulatively experienced in nurse and GP work. The interview guide primarily used open ended questions to explore (i) the cooperation between GP and patient, (ii) the role of other health care professionals in managing the patient, (iii) the course of the patient’s last hospitalization and discharge and (iv) the expected future needs of the patient. The interview guide was influenced by the multidisciplinary background of the research team. The team held meetings during the project period to evaluate ongoing interviews and insights gained.

Semi-structured interviews were conducted with twenty recruited GPs (45% male) aged between 27 and 65 years (M = 43.5 years, average 45.1 years). Recruited GPs primarily provided their services from group practices (*N* = 19) and had 800–1600 enlisted patients (M = 1200, average 1165). Three GPs were locum tenens. Interviews generally lasted 1 h (32 min – 1 hour 22 min) and were carried out between October 2019 and January 2020. Interviews were conducted face-to-face with the GP in his or her practice. One GP interview was, however, conducted at the GP’s home office. All interviews were audio recorded.

### Data analysis

Interviews were transcribed verbatim, de-identified and imported into the social research software Nvivo (version 12) for data analysis.

**Fig. 1:**
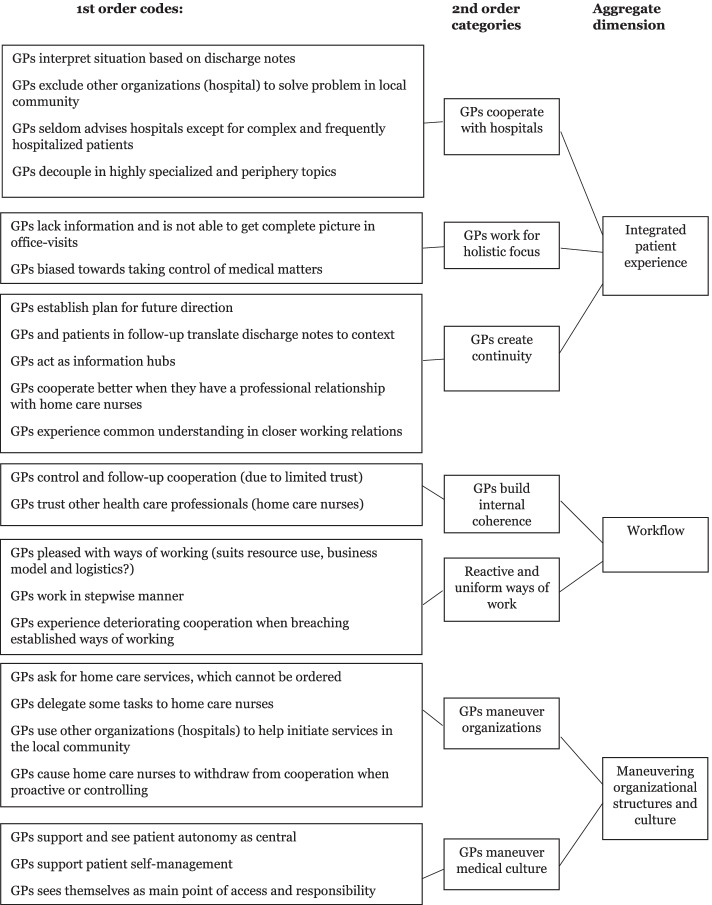
Data structure

Data analysis was performed by the first author (HB) under the supervision of one of the co-authors (OØ) who is experienced in the selected methodology. Initial analysis was performed by the first author (HB). This consisted of coding each interview separately, first order codes being revealed from interview objects, words and phrases [28, 29]. Interviews were reread a number of times and meetings were held between HB, AM, MS, and OO to discuss and agree on emerging findings. Codes that were in essence similar were categorized into the same first order concept (Table 1). We also began uncovering and mapping connections between them whilst carrying out first-order coding. This first stage of coding is informant-centric. There was therefore no pre-defined coding tree. A broad and open approach was, however, applied to leadership and to the questioning of what actions GPs take to get things done when interacting with patients and other health care professionals. The theoretical groupings that emerge from this process represent second-order themes (Table 1) that, in contrast to first-order concepts, are researcher-centric [26]. Identification of aggregate dimensions from second order themes (Table 1) enables the development of a theoretical framework that builds on the findings of our data structure (Fig. 1).

**Table 1:** Exemplary quotations and the 1st order concepts, 2nd order themes and aggregate dimension identified from data analysis

**Aggregate dimension: Process of creating an integrated patient experience**	**2nd order themes**
**1st order concepts with exemplary quotations and actions**	
**GPs interpret situation based on discharge notes** - It’s not always as easy as this. Sometimes I need to call and ask them to send (…) an unfinished discharge note so that I can understand what’s been done.	**GPs cooperate with hospitals**
**GPs exclude other organizations (hospital) to solve problem in local community** - There is not much more they can do, there are no more investigations to carry out. So, it is (medical condition) management supervised by me.	**GPs cooperate with hospitals**
**GPs seldom advise hospitals except for complex and frequently hospitalized patients** - Then, I write that if they cannot do anything with it now, I think it will be ok and that he can leave and go home and be called on later for follow-up.	**GPs cooperate with hospitals**
**GPs decouple in highly specialized and periphery topics** - Dialogue is often from them to me. (…) I don’t have much to contribute when hospitalized. Then, responsibility of treatment is transferred to the hospital.	**GPs cooperate with hospitals**
**GPs lack information and is not able to get complete picture in office-visits** - I only see him in the office setting. (…) So, it is obvious that he may have needs that I don’t see, and that doesn’t come up during our conversations.	**GPs work for holistic focus**
**GPs biased towards taking control of medical matters** - I messaged home care nurses, informing them that now we will do it this way, and that they can provide the medicine (…) until it comes from the pharmacy.	**GPs work for holistic focus**
**GPs establish plan for future direction** - They don’t know what to do. So, that is why they contacted me now. We have established a plan now, and then we will have to see if it goes well (…).	**GPs create continuity**
**GPs and patients in follow-up translate discharge notes to context** - We summarize and read what’s been done at the hospital, and they can ask questions if there are any from the patient’s perspective.	**GPs create continuity**
**GPs act as information hubs** - Home care nurses are my extended arm to the patient, and (…) alert me if anything is needed. Thus, it is my responsibility to be a patient coordinator.	**GPs create continuity**
**GPs cooperate better when they have a professional relationship with home care nurses** - For this patient I know the people who provide him services, therefore it is easier to communicate and agree on things.	**GPs create continuity**
**GPs experience common understanding in closer working relations** - (…) I don’t need to use the telephone much in communication with home care nurses as they understand the patient’s complexity and needs.	**GPs create continuity**
**Aggregate dimension: Process of workflow**	**2nd order themes**
**1st order concepts with exemplary quotations and actions**	
**GPs control and follow-up cooperation (due to limited trust)** - Then, I guess I secure my work more (…) and, if highly important, ask them for a response and make a reminder for myself.	**GPs build internal coherence**
**GPs trust other health care professionals (home care nurses)** - Because they see her/him often, they have a greater ability to assess how s/he is doing than me who doesn’t see her/him that often.	**GPs build internal coherence**
**GPs pleased with ways of working (suits resource use, business model and logistics?)** - Yes, because I know what’s going on up there, and if s/he needs help with anything, I may be able to contribute, If I get to know we can find solutions.	**Reactive and uniform ways of work**
**GPs work in stepwise manner** - No, there is no need (for meetings). We talk sometimes (telephone) at the beginning, when things need to be clarified, otherwise everything has been digital.	**Reactive and uniform ways of work**
**GPs experience deteriorating cooperation when breaching established ways of working** - It may be that home care nurses are involved with other GPs who take less responsibility than I do, but I think it’s wrong that I should have an even bigger workload because I try to do a good job.	**Reactive and uniform ways of work**
**Aggregate dimension: Process of maneuvering organizational structures and culture**	**2nd order themes**
**1st order concepts with exemplary quotations and actions**	
**GPs ask for home care services, which cannot be ordered** - When (…) discharged from the hospital I experienced her/him as being still very worn out, so I sent a digital message asking them to adjust the care services.	**GPs maneuver organizations**
**GPs delegate some tasks to home care nurses** - S/he had a permanent urinary catheter and I advised it to be changed. So, they have changed it every other month or so.	**GPs maneuver organizations**
**GPs use other organizations (hospitals) to help initiate services in the local community** - I hope s/he can have a higher level of care. I hope the hospital have taken care of that now. Because it’s much harder for me to get it done.	**GPs maneuver organizations**
**GPs causes home care nurses to withdraw from cooperation when proactive or controlling** - I have the impression that if I’m not that proactive, the home care nurses will be more attentive, but it would be nice to have some communication back and have a dialogue (when I’m proactive).	**GPs maneuver organizations**
**GPs support and see patient autonomy as central** - Thus, we don’t do much other than take care of him/her, sort of. But we try to make him/her accountable for his/her own health.	**GPs maneuver medical culture**
**GPs support patient self-management** - No, patients are their own coordinators as long as they are “reasonably well functioning”.	**GPs maneuver medical culture**
**GPs see themselves as main point of contact and responsibility** - I think it is nice that everything is in one place and that responsibility is held by as few as possible.	**GPs maneuver medical culture**

## Results

We, from our data, identified 23 first order concepts and seven second order concepts (Table 1). We subsequently identified that GPs provide IC for elderly multimorbid patients through three processes. These are (A:) the process of creating an integrated patient experience, (B:) the workflow process and (C:) the process of maneuvering organizational structures and medical culture (Fig. 1).

These three processes are presented in the following with findings from our emerging data structure presented in Fig. 1, translated verbatim extracts that show how we arrived at our findings being presented in Table 1 and the appendix (Table A1).

### A: process of creating an integrated patient experience

The 2nd order theme “Process of creating an integrated patient experience” captures the context-near 1st order concepts that the GPs are involved in and use, to acquire an understanding of the current situation, to adjust interventions to individual holistic patient needs and create continuity for the patient.

We see, in our data, that GPs primarily participate actively in critical situations or changes where there is a great deal of activity around the patient, e.g. shortly before and after patient hospitalization. Digital communication is in place. GPs are, however, viewed as possessing the “complete picture” and the most up-to-date information, especially where information is old, not digitalized, or tacit (Fig. 1).

The 1st order concept “GPs establish plan for future direction” (Fig. 1) shows GPs investing effort in communicating clearly to ensure “all know”. GPs often consider that their assessment and digital correspondence represents the true medical situation, and also the management plan for the foreseeable future.

Maybe I need to be more careful, to be even better at writing health care records, so everyone can understand what I write*.*

GPs express that they, through receiving and transferring information, act as a central information hub for the patient. They see themselves, in situations where opinions and views of different medical specialists diverge, as being responsible for prioritizing and setting directions for treatment in clinical day-to-day practice. GPs apply a pragmatic approach. They, when decisions are to be made and tasks are to be carried out within their own core area of competence, balance their knowledge of the patient’s history, their own professional experience, and the views of other health care professionals (e.g. hospital specialists). GPs often, however, leave decisions and tasks to the specialist where treatment and follow-up involve highly specialized decisions or equipment. GPs commonly, when needed, communicate digitally or by telephone with specialists for advice.

Hospital discharge notes exert an influence on GPs and patients. The GP and the patient try however, during follow-up, to adapt their course of action to the patient’s history and most likely future, within the possibilities and limits set in the hospital discharge note.

The 1st order concept “GPs cooperate better when they have a professional relationship with home care nurses” shows that closer relationships and a better understanding develops between GPs and the patient’s network of nurses, where they hold meetings or correspond frequently. GPs experience that digital cooperation can improve after physical meetings.

The 2nd order theme of “GPs work for holistic focus” and an understanding of the patient’s situation, shows that GPs sometimes miss information in complex cases, and are sometimes biased towards the medical aspects in patient care (Fig. 1). Some GPs worry that all the needs of patients cannot be uncovered during practice visits, and that they can only be uncovered in the environment in which the patient lives and experiences their life. A limited number of GPs were concerned that structured digital text-correspondence offers fewer opportunities for “talk” that can uncover tacit problems.

Yes. I think we had more meetings before, if someone were troubled, to try and set a direction for treatment and follow-up*.*

Though GPs have a pragmatic approach and sometimes reverse decisions made in specialist health care, the 2nd order theme “GPs cooperate with hospitals” represents the consistent finding that the opinions and directions of specialist care providers are a central element in the GPs understanding of the patient’s situation and future pathway. The first order concept “GPs interpret situation based on discharge notes” shows that GPs hold strong opinions on the quality of discharge notes, missing discharge notes also commonly resulting in reactive behaviors such as calling hospitals or other actors in the health care system.

GPs conversely, however, play a less central role in the hospital treatment of a patient than the hospital plays in GP treatment. We observe that GPs only occasionally set the direction when patients are hospitalized, and that this is often when the patient case is complex or where the patient has been frequently hospitalized in the recent past.

### B: process of workflow - contributing to internal coherence of services and working in a stepwise manner

Interviews identified the securing of internal coherence in health care service provision to be a central element of a GP’s job. The interviews also identified that most GPs use a reactive and stepwise approach to solving ongoing and emerging problems. These two 2nd order themes together make up the second order aggregate dimension “Process of workflow”.

GPs express trust in other health care professions, but want to monitor and be informed about ongoing situations and work processes, to make sure they are implemented and to follow up quality. GPs rely on digital tools in this, unless the complexity of the situation requires telephone calls or physical meetings. Increased trust in and task sharing (patient follow-up, drug tapering) with the home care nurse were occasionally observed. This was, however, limited to situations in which the GPs had in-depth knowledge, and where the nurse had a thorough knowledge of the patient’s life and situation. Some GPs had limited trust in the digital system and created control mechanisms to ensure that important tasks had been executed by home care nurses.

In a way I feel I get more control, but at the same time you cannot always trust that what you write down will be done*.*

The 2nd order theme “reactive and uniform ways of work” streamlines workflows and ensures that the work is carried out efficiently. The 1st order concept “GP works in stepwise manner” captures that day-to-day work cooperation and correspondence primarily consist of digital text messages between GPs and home care nurses. The next steps that are to be taken by the message sender or recipient are communicated and discussed in these messages. Higher levels of communication are, however, required when things become more complicated. Digital correspondence is commonly limited to changes in drug treatment or more elementary clinical measures. GPs say that they use the telephone and initiate meetings in more complex cases. Home visits are only carried out occasionally in response to semi-acute problems. GP participation in proactive activities or planning commonly occurs in an “proactive on reactive” pattern. An event triggers a system action, after repeated visits to hospital or the local emergency care room.

GPs consider the digital system to be a flexible way of updating colleagues, of discussing and managing drug lists, of “staying in the loop” and monitoring the patient’s situation. This is covered by the 1st order concept “GPs pleased with ways of working”.

As GP answers digital requests in batches, digital communication is not in real-time. This results in a potentially high number of short and fluid partnerships between a GP and different home care nurses. This requires communication to be rigid and structured, so that everyone can understand it. Digital communication is primarily text based. The lack of flexibility of this communication form may therefore lead to a monotonous communication.

A central finding of the second identified aggregate dimension “Process of workflow”, is that most GPs ultimately use the digital communication system in a similar rigid and monotonous way, the way that they work being characterized by a “step-wise” and “proactive on reactive” approach.

### C: process of maneuvering organizational structures and medical culture - positioning the GP role in relation to patients and the health care system

Much of the hardship experienced by GPs when trying to set direction outside and beyond their own organization, is captured by the aggregate dimension that describes GPs maneuvering organizational structures and medical culture. GPs are efficient when setting the direction of medical aspects across organizations. Examples include changes in medication and clinical measurements. GPs are, however, not as efficient in less medical issues such as initiating physiotherapy at home, short term stays in nursing homes or other tasks that are less strongly linked to the GP role. One GP said that it was easier when hospitals administered the admission of patients to nursing homes on discharge from hospital. This implies that hospitals have greater access to nursing homes than GPs. GPs sometimes, furthermore, use their medical authority to hospitalize patients, to help overcome organizational hindrances so that patients receive health care services from the municipality after hospital discharge.

The GP can, in other situations, be positioned at the opposite side of the spectrum of power. One GP had experienced home care nurses withdrawing from digital cooperation when the GP intervened actively, exercised too much leadership or was too controlling. We therefore conclude from the 1st order concept “GP causes home care nurses to withdraw from cooperation when proactive or controlling”, that GPs must be careful and follow established rules of cooperation to avoid other stakeholders withdrawing from task implementation.

Finally, findings revealed that medical culture affects GPs’ perspective on IC. The GPs interviewed frequently raised the importance of patient autonomy, and expressed their support for patient self-management. We frequently observed GPs seeing themselves as the main point of contact, the “first responder” and the coordinator of the overall medical services received by a patient. This, taken with GPs commonly expressed aim that patients are handled “in the municipality”, leads us to conclude that GPs in this cohort see themselves as gatekeepers to the wider health care system. This is captured in the 1st order concept “GPs see themselves as main point of contact and responsibility”.

I think it is nice that everything is in one place and that responsibility is held by as few as possible*.*

**Fig. 2:**
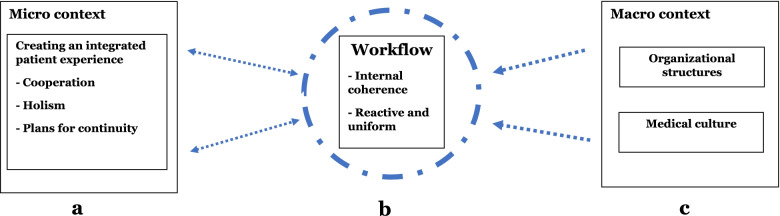
GPs’ involvement in collective efforts in IC: Aims of cooperating well, being holistic and planning for continuity (**a**) within the established way of working (**b**) and influence of organizational structures and medical culture (**c**)

To summarize, results from interviews show that GPs play a central role in a patient’s health care team and that GPs, through primarily focusing on the patient and the micro-context, consider that IC is provided when patients experience cooperation, holism and continuity in service provision (Fig. 2a). The 2nd order aggregate dimensions identified by our analysis, demonstrate the challenges that confront GPs who aim to exercise leadership across organizations (Fig. 2). These challenges arise from the creation of an integrated patient experience in cooperation with different health care professionals (Fig. 2a), from constraints resulting from the step-wise uniform way of working (Fig. 2b) and the requirement that the GP acts in accordance with the macro-context, which consists of organizational structures and the prevailing medical culture (Fig. 2c).

## Discussion

By exploring what type of leadership actions GPs adopt in collaboration with patients and other health care professionals to provide IC, we identify that the collaboration between GPs, patients and other health care professionals in this municipality can be characterized as DL.

**Fig. 3:**
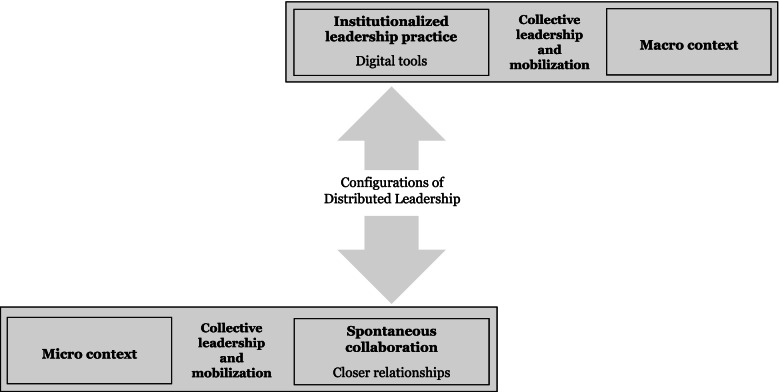
Location of collective leadership and observed configurations of DL

Digitally facilitated correspondence between health care professionals in this municipality frequently bears similarities with the configuration of “institutionalized leadership practice” observed in DL [17]. Structured and formalized tasks and functions have, in this, resulted by design [17] or from “planful alignment” [34]. Our findings show that in this configuration, collective leadership commonly resides in collective initiatives and efforts mobilized from the digital solutions in use and the macro-contextual environment of organizational structures and medical culture (Fig. 3). GPs primarily accommodate and observe “the whole” from the digital space, balancing their roles as both leaders and followers. Leadership is, however, not easily observed, as many of the collective tasks in this configuration are of a managerial nature.

Literature on how DL is implemented or operates in health care is scarce [35] and only a few studies have explored DL in the primary care or municipality setting [36]. In accordance with the general literature on IC, the process of care integration in this municipality relies on effective digital tools for information sharing [37, 38]. However, findings from this study suggests that digital tools are not fully utilized but adapted to suit the established workflow and context (Fig. 2), facilitating GP participation in multiple parallel work groups which is essential in DL. Furthermore, findings indicate that rigid and structured digital communication minimize challenges related to role overlap and role ambiguity identified in a previous study on DL in a community mental health context in Canada [39].

Taken as a whole, findings from this study show that the leadership actions GPs adopt to collaborate with other health care professionals in this municipality can be characterized as DL and contribute to IC. However, the digitally facilitated and institutionalized configuration of DL frequently identified in this municipality primarily contribute to service integration at the organizational level. It is worth noting that this finding is in line with findings from a recent study by Salmon et al. [40]. In this study DL was found to promote streamlined service provision and to facilitate service and system-level integration in an emerging network of integrated youth health care centers in a municipality setting in Canada [40].

GPs, when they occasionally take more action to affect the direction of collective efforts in the provision of IC, commit to closer relationships with other health care professionals, act out more interpersonal roles as figureheads in the solving of complex problems, and become enabled to both monitor and disseminate “the complete picture” of information in interaction with other health care professionals. Such situations can be equated to configurations of “spontaneous collaboration” in DL, groupings of individuals from different organizational levels pooling their expertise for the duration of the task and then disbanding [17]. Collective leadership resides, in this configuration, in the micro-contextual environment of the patient, collective efforts being mobilized to bring about home visits or in-office meetings, which are initiated by GPs, peers, or other health care professionals (Fig. 3). This configuration is, however, only observed occasionally, exists only within smaller work groups disconnected from the wider organizational context, move spontaneously only after being collectively initiated in a “proactive on reactive” way in response to a “crisis”, and commonly apply only to a limited domain related to the problem which caused the call for collective efforts.

Findings complement previous studies on DL, showing that roles and responsibilities are fluid, temporary and influenced by the wider organizational context [41, 42], and that strong interpersonal relationships is a contextual factor that promotes DL [42]. Consistent with discussions on the macro-context in this study (Fig. 2c), previous studies have established that organizational factors, professional roles, and values influence the distribution of leadership in health care [15, 42–44].

Of the two identified configurations of DL, “spontaneous collaboration” intuitively seems to be better suited to the achievement of individual and complex IC goals. Our findings show, however, that there are many factors within the micro and macro-context that affects the form of collective leadership observed (Fig. 2). Applying efficient institutionalized ways of “planful alignment” one or more times before moving on to more effective but resource and human capital-intensive methods of “spontaneous collaboration”, may serve to achieve quality goals and the limiting of the resources used in this health care system context where personnel scarcity is described as being the limiting factor [22]. However, DL as social process and construct depends on continuity and disappears if there is lack of follow-up, meeting points or information sharing between people (Fig. 2b). Findings from this study restates the importance of relationship building and context in DL [45].

If the aim of establishing DL is to improve patients experience of IC, GPs’ and other health care professionals’ understanding of what IC is must be uncovered as a strong GP focus on continuity and cooperation may primarily serve IC as a “top-down process” at organizational levels [46]. The macro-context may furthermore benefit organizational goals, but at the cost of multimorbid patients’ needs for swift and individualized measures [5, 47].

Our findings suggest that when the capacity of digital tools is limited or there is rationing of health care personnel, then collective leadership actions fall into “institutionalized” and “preplanned” ways of working, constrained by the shared agency of the macro-context. It is expected, in the theoretical conceptualization of DL in health and social care, that synergies from DL arise when concertive actions and conjoint agency interact [15]. Theorizing opens for discussions on whether a strongly held “conjoint agency”, larger groups or groups with limited resources are predisposed to establishing institutionalized and “preplanned” ways of working (Fig. 3). It also opens for a discussion of whether interactions between “concertive actions” and “conjoint agency” can have negative synergies that make DL rigid and inflexible. We are not aware of researchers discussing negative synergies in DL. We are also unaware of discussions of the need for health care professionals or patients to agree on a more contextualized “conjoint agency” disconnected from the wider mission or purpose of the health care organization in DL.

This study has some limitations. Seeing leadership as a social construct that emerges within groups, may limit the ability of the study to uncover what health care professionals do not do. This may also limit the examination of the role of individual characteristics, such as the professional power and personal interests of GPs. Recruited patients may have been those at “the top of the mind” of the GP or their secretary, those who are easy to approach due to upcoming scheduled appointments, those expected to participate constructively or very complex cases from the perspective of IC. Recruitment was limited to one patient per GP to reduce this bias. As the first author (HB) has experience from work as a GP in the municipality where the research project was carried out, there is a possibility of social desirability bias in GP interviews. Consulting the multidisciplinary research team during analysis is expected to have minimized this bias.

## Conclusion

The results of this study shows that health care professionals who aim to facilitate DL in IC should focus on recognizing and unifying the multiple and shifting contexts experienced by patients, be relational with other health care professionals and master several ways of cooperating across organizational borders. In this municipality DL was predominantly observed as institutionalized practice and “planful alignment” contributing to organizational integration and coordination. Achieving “higher forms” of DL, in which collective leadership and efforts emerge as social processes and parts of living systems connected to a patient context, challenges current ways of working, and the application of digital tools, use of health care personnel and resource use.

## Supplementary Information

**Additional file 1.**

## Data Availability

The datasets generated from the current study are not publicly available due to reasons of confidentiality. Additional knowledge of the de-identified data is available from the corresponding author on reasonable request.

## Abbreviations

DL: Distributed leadership
IC: Integrated care
GPs: General practitioners
