# Supplementary material for: General practitioners’ perceptions of distributed leadership in providing integrated care for elderly chronic multi-morbid patients: a qualitative study
Source: BMC Health Serv Res. 2022 Aug 25;22:1085. doi: 10.1186/s12913-022-08460-x (PMC9404619; doi:10.1186/s12913-022-08460-x)
Supplement: Supplementary file 1 — Additional file 1. [file 12913_2022_8460_MOESM1_ESM.docx]

Appendix: Translated verbatim extracts from interviews with GPs (Table A1)

| **Representative quotation (1st order categories)** | **Second order Themes** | **Overarching dimension** |
| --- | --- | --- |
| **GPs interpret situation based on discharge notes**  - This time there were no problems. But it’s not always as easy as this. Sometimes, concerning other patients in (in general), I need to call and ask them to send digitally or telefax, even an unfinished discharge note so that I can understand what’s been done.  - Information concerning the (name of department) part of the stay was lacking. I did not get much information about what they were thinking about the (condition) and what should be followed-up.  - Yes, it was unclear to me what they really meant. And I received that afterwards … First received a discharge note from (name of regional hospital) without information on this, and then I got a digital message afterwards.  - Discharge notes, typically they arrive directly on discharge. That makes follow-up easier. | **GPs cooperate with hospital** | **Process of creating an integrated patient experience** |
| **GPs exclude other organizations (hospital) to solve problem in local community**  - It was reasonable to discharge the patient when they did. It is here, at the municipality level, we need to find out how to optimize this, because it is not a hospital task.  - They said that there is not much more they can do, there are no more investigations to carry out. They will not do any more. So, it is (name of medical condition) management supervised by me | **GPs cooperate with hospital** | **Process of creating an integrated patient experience** |
| **GPs seldom advise hospitals except for complex and frequently hospitalized patients**  - Then, I write that if they cannot do anything with it now, I think it will be ok and that he can leave and go home and be called on later for follow-up (…) But, this is primarily for elderly patients that do not want to be hospitalized, but where we think it is necessary for some tasks such as blood transfusions.  - And then, I wrote a bit of the history and how it has been at the time of hospitalization, and that I have discussed the problems with other people in the hospital before to avoid that they start something new now. And then I asked for, when they have finished this, if they could consult the (name of) division. So, that was the real reason I hospitalized him. Because I don’t think there is anything wrong with his (part of body), but we must make sure. Because, I really wanted to hospitalize him in the (name of) division, but they would have said that he needed to be (name of department) assessed, as they call it, and sent him to the hospital anyway | **GPs cooperate with hospital** | **Process of creating an integrated patient experience** |
| **GPs decouple in highly specialized and periphery topics**  - We did not have any communication as long as the patient was hospitalized. They took care of the treatment, and then I got the discharge notes on the same day the patient was discharged.  - And then there is (name of organ) treatment and changes to (name of treatment) that I don’t always have an overview of, as it is primarily the (name of department)-doctor who writes prescriptions. However, I sometimes get inquiries from home care nurses concerning medications, and then I must search to see if the patient has this or that treatment  - Dialogue is often from them to me. They ask me if they have questions. I don’t have much to contribute when hospitalized. Then, responsibility of treatment is transferred to the hospital Doctor. | **GPs cooperate with hospital** | **Process of creating an integrated patient experience** |
| **GPs lack information and is not able to get complete picture in office-visits**  - Yes. I think we had more meetings before, if someone were troubled, to try and set a direction for treatment and follow-up.  - I only see him in the office setting. 25 minutes once a month maybe. So, it is obvious that he may have needs that I don’t see, and that doesn’t come up during our conversations | **GPs work for holistic focus** | **Process of creating an integrated patient experience** |
| **GPs biased towards taking control of medical matters**  - I messaged home care nurses after meeting with the patient, informing them that now we will do it this way, and that they can provide the medicine to the patient until it comes from the pharmacy, so that we effectuate it straight away (…). Or, we say that it is less of a hurry, and that we can wait until it comes from the pharmacy.  - I received a list from the pharmacy that did not look like mine nor the one from (health care institution). Therefore, I contacted the home care nurses who sent me their list. I looked at the one from the (health care institution because it was the most recent, tried to update from this, sent it back and got a new list back that was much more in line with the list I had, and approved this.  - But sometimes it’s too much. Very easy for home care nurses to send some request about something *(non-medical)* that I don’t need to know anything about  - I have contact with home care nurses sometimes. I can send them a (digital) message that they should consider it, but I think it comes from the hospital in his case.  - Now, they have sent some… They want information on diagnoses and such (*administrative character*), but in other ways it has been functioning well. | **GPs work for holistic focus** | **Process of creating an integrated patient experience** |
| **GPs establish plan for future direction**  - Maybe I need to be more careful, to be even better at writing health care records, so everyone can understand what I write”.  - And I write it instead, so it’s written down in addition. I think that is the best thing about it (use of digital correspondence).  - They don’t know what to do. So, that is why they contacted me now. We have established a plan now, and then we will have to see if it goes well, and if not, they will need to contact me again. | **GPs create continuity** | **Process of creating an integrated patient experience** |
| **GPs and patients in follow-up translate discharge notes to context**  - He says what’s been said at the hospital, and what his experience was. And there is some like, you know, if you’re soon (n) years old there can be many misunderstandings.  - We summarize and read what’s been done at the hospital, and they can ask questions if there are any from the patient’s perspective. | **GPs create continuity** | **Process of creating an integrated patient experience** |
| **GPs act as information hubs**  - It is the home care nurses who are my extended arm to the patient, and it is home care nurses who alert me if anything is needed, like a home visit or similar. Thus, it is my responsibility to be a patient coordinator.  - After we started digital communication, I receive digital communication from home care nurses, often daily about patients. Not just her/him but all patients like her/him.  - Yes. Emergency care have called, but home care nurses call often. | **GPs create continuity** | **Process of creating an integrated patient experience** |
| **GPs cooperate better when they have a professional relationship with home care nurses**  - For this patient I know the people who provide him services, therefore it is easier to communicate and agree on things  - There were three home care nurses there, and then we get to know each-other so that we speak very freely about the patient’s problems. Yes, and when we communicate together via digital communication or by telephone, we have a completely different understanding about what’s going on. That is really wise.  - Communication has changed after we held that meeting. | **GPs create continuity** | **Process of creating an integrated patient experience** |
| **GPs experience common understanding in closer working relations**  - I experience that home care nurses, they also know the patient well and do make what I consider to be good assessments of the patient, so that I think digital communication with home care nurses works well because we have a common understanding of the patient. Therefore, I don’t need to use the telephone much in communication with home care nurses as they understand the patient’s complexity and needs.  - You know, those who are involved with this patient understand that s/he makes a lot of noise about things that are not that dramatic. | **GPs create continuity** | **Process of creating an integrated patient experience** |
| **GPs control and follow-up cooperation (due to limited trust)**  - If it concerns home care nurses in general, then I lack knowledge on departments, who’s who, and who’s the patient’s primary contact. Then, I guess I secure my work more by making clear agreements via digital communication, writing clearly what I want them to follow-up on and, if highly important, ask them for a response and make a reminder for myself in case I have not heard from the home care nurse.  - In a way I feel I get more control, but at the same time you cannot always trust that what you write down will be done. In her/his case it has been all right.  - I received confirmation (on multi-dose packaging from the pharmacy), so then you think that this information is out. But if you’re going to be sure it will depend on the patient and his/her function. (…) I hope and believe that home care nurses feel some ownership and responsibility, that they also feel responsible for medical treatment. However, sometimes you’re a bit unsure whether they feel that, as you feel some lack of control of the medical treatment yourself.  - It depends a lot on the one (home care nurse) who provides the services. | **GPs build internal coherence** | **Process of workflow** |
| **GPs trust other health care professionals (home care nurses)**  - Because they see her/him often, they have a greater ability to assess how s/he is doing than me who doesn’t see her/him that often.  - I think the home care nurses are a professional actor in this, and can more easily see if patients have medical needs.  - The home care nurses are very active. (…) If there is anything, something that is not right, they call the hospital directly to clarify.  - (At discharge) there was more dialogue with home care nurses, and then home care nurses were told to contact me based on criteria, e.g. after tapering (name of medical treatment) they should contact me on that topic. (…) So, this was written in the discharge notes, so then I knew, and I knew that she (home care nurse) would follow-up and contact me when it happens. | **GPs build internal coherence** | **Process of workflow** |
| **GPs pleased with ways of working (suits resource use, business model and logistics?)**  - Yes, because I know what’s going on up there, and if s/he needs help with anything, I may be able to contribute, I get to know. Then, if I get to know we can find solutions.  - Yes, I think digital communication is very clever. You receive messages, and you have this continuity with the patient and how s/he is doing and what they think about the situation. I think that is great.  - When I have not heard from her/him, which is very infrequent. Then I may send a message to ask if home care nurses have visited and how her/his situation is. That can be a solution that works well, and then we have a three-way dialogue where s/he feels seen and taken care of. | **Reactive and uniform ways of work** | **Process of workflow** |
| **GPs work in stepwise manner**  - No, there is no need (for meetings). We talk sometimes (telephone) at the beginning, when things need to be clarified, otherwise everything has been digital.  - I send the home care nurses digital correspondence if there is something they need to know.  - I usually send a report to the home care nurses after talking to the patient, because it regularly includes some changes or other updates concerning the patient. Today I received a message from home care nurses before the patient’s appointment.  - I commonly send a message after meeting the patient if there are any changes, so that I am sure that the information is passed on. As I know the patient cannot transmit the information. And she also sends information to me before appointments if there is anything special she has been thinking of, to make sure the information reaches me.  - If it is acute, we use the telephone, and if it can wait, then we use digital communication.  - I use digital communication with the hospital, or I use the telephone if I need help there and then.  - Today, the daughter/son, the patient and I talked about holding a meeting on what s/he thinks about where s/he wants to live.  - No, then I made a home visit, and I said; “OK, let’s take away 5 mg tablets (…) and change for 10 mg and aim for a daily maximum dosage of 40 mg to be administered around the clock.  - If this was a less complex patient, who maybe just had one problem. And you were just thinking of giving one message that did not result in that many possible questions and needs for clarifications, then I think a digital or written message would be just as good.  - No, if in a hurry, they know that they need to telephone, contact directly or contact the emergency room if we cannot be reached. | **Reactive and uniform ways of work** | **Process of workflow** |
| **GPs experience deteriorating cooperation when breaching established ways of working**  - It may be that home care nurses are involved with other GPs who take less responsibility than I do, but I think it’s wrong that I should have an even bigger workload because I try to do a good job. | **Reactive and uniform ways of work** | **Process of workflow** |
| **GPs ask for home care services, which cannot be ordered**  - When s/he was discharged from the hospital I experienced her/him as being still very worn out, so I sent a digital message to the home care nurses asking them to adjust the care services at home, for her/him and the husband/wife that is. | **GPs maneuver organizations** | **Process of maneuvering organizational structures and culture** |
| **GPs delegate some tasks to home care nurses**  - S/he had a permanent urinary catheter and I advised it to be changed. So, they have changed it every other month or so.  - The (name of medicine) variant also. I explained to them how they should adjust (the medicine), up or down, depending on (measurements). And they have confirmed that they will do it. The patient has also provided written consent that home care nurses can do it and are responsible for this medicine. | **GPs maneuver organizations** | **Process of maneuvering organizational structures and culture** |
| **GPs use other organizations (hospitals) to help initiate services in the local community**  - I hope s/he can have a higher level of care. I hope the hospital have taken care of that now. Because, it’s much harder for me to get it done. | **GPs maneuver organizations** | **Process of maneuvering organizational structures and culture** |
| **GPs causes home care nurses to withdraw from cooperation when proactive or controlling**  - I have the impression that if I’m not that proactive, the home care nurses will be more attentive, but it would be nice to have some communication back and have a dialogue (when I’m proactive). | **GPs maneuver organizations** | **Process of maneuvering organizational structures and culture** |
| **GPs support and sees patient autonomy as central**  - No, s/he had those services before s/he chose not to have them.  - In her case, s/he is good at taking care of her health. Thus, we don’t do much other than take care of her, sort of. But, we try to make her accountable. | **GPs maneuver medical culture** | **Process of maneuvering organizational structures and culture** |
| **GPs support patient self-management**  - No, patients are their own coordinators as long as they are “reasonably well functioning”.  - Yes, if the patients wants, s/he can handle everything.  - It can be hard to uncover needs, because I think she wants to manage on her own. | **GPs maneuver medical culture** | **Process of maneuvering organizational structures and culture** |
| **GPs see themselves as main point of contact and responsibility**  - I think it is nice that everything is in one place and that responsibility is held by as few as possible  - I’m the Doctor he needs most often. So, if his/her health deteriorates I will be the one s/he sees first  - I have the medical responsibility for this patient. It’s home care nurses who are my extended arm reaching out to the patient (…) It is my responsibility to be the patient’s coordinator. | **GPs maneuver medical culture** | **Process of maneuvering organizational structures and culture** |

**Table A1: Translated verbatim extracts from interviews with GPs**
